# Mixing of Dead Sea and Red Sea waters and changes in their physical properties

**DOI:** 10.1016/j.heliyon.2020.e05444

**Published:** 2020-11-07

**Authors:** Abdelaziz Khlaifat, Mufeed Batarseh, Khalid Nawayseh, Jamal Amira, Emad Talafeha

**Affiliations:** a Petroleum and Energy Engineering Department, American University in Cairo, Cairo, Egypt; b Abu Dhabi Polytechnic, Abu Dhabi, United Arab Emirates; c Arab Potash Company, Jordan; d Mutah University, Chemistry Department, PO Box 7, Karak, Jordan

**Keywords:** Environmental science, Geochemistry, Earth sciences, Hydrology, Oceanography, Physical, Temperature, pH, Level, Dissolved oxygen, Density, Salinity and viscosity, Dead sea, Red Sea, Mixing

## Abstract

The present work emphasizes on the changes in the Red Sea and Dead Sea mixed waters physical properties including: temperature, pH, dissolved oxygen, density, salinity and viscosity. It focuses on the impacts of changes in mixed water quality on the Dead Sea ecosystem and the current industrial activities. The pilot project site consisted of six water ponds (tanks) located next to Arab Potash Company point of intake about 100 m south of the Dead Sea shores. The Red Sea - Dead Sea water mixing was controlled and done based on the expected mixing ratios between Red Sea and Dead Sea waters to mimic the potential actual situation associated with Red Sea – Dead Sea project conduit.

All measured properties of mixed water bodies in tanks 1 to 5 tend to behave differently from similar Dead Sea water (tank 6) properties. The properties variations depend on the rate of diluting the Dead Sea water by Red Sea water and rejected brine. The least altered physical properties were observed when the Red Sea concentrated brine was added to the Dead Sea water (tank 5). The obtained results show that transferring Red Sea water to Dead Sea would lead to dilution of Dead Sea brine and affects significantly the investigated mixed water physical properties. Water mixing project is expected to cease the halite precipitation phenomenon due to the development of stratification in the Dead Sea and halite dissolution.

Based on the industrial needs for the Dead Sea brine with its current physical properties, it is recommended to add rejected brine only to the Dead Sea due to its minimal effect on physical properties variations.

## 1 Introduction

The Dead Sea is a closed lake and the riparian countries are Jordan, Palestine and Israel [1, 2, 3]. Its water level reported at 431 m below sea level (bsl) in August 2018, while, it was 416 m bsl in 2003 (Oren et al. [4]; Khlaifat et. al., [1, 2, 5, 6]). This decline in water level confirmed by the hypsometric curve of Neev and Emery [7], Dead Sea covered an area of 950 km^2^ at 397 m bsl. The Dead Sea area had shrunk to about 780 km^2^ after 1976 and its volume decreased from 150 km^3^ to 135 km^3^.

The Dead Sea is the largest hypersaline water body on the planet. It has a salinity of 340 g/L (Khlaifat et.al., [2, 5]; Herut [8]). Magnesium (Mg 46 g/L) is the most dominated cation in Dead Sea water and it's called Magnesia Sea, followed by Sodium (Na 36.5 g/L), Calcium (Ca 17 g/L) and Potassium (K 7.8 g/L), while, Chloride (Cl 225 g/L) is the main anion followed by Bromide (Br 5.6 g/L). Carbonate (CO_3_^−2^) and sulfate (SO_4_^−2^) are minor components in Dead Sea water (Gavrieli, [9]). Neev and Emery [7] studied the composition of the carbonate system in Dead Sea. They observed two mineral precipitation events during the summer, they called these events “brine whitening” which resulted in gypsum (CaSO_4_.2H_2_O) to aragonite (CaCO_3_) weight ratio greater than 3.14. The major minerals in Dead Sea were aragonite (CaCO_3_), anhydrite (CaSO_4_), and halite (NaCl), and their concentrations found saturated to oversaturate (Gavrieli, [9]; Katz et al., [10]; and Krumgalz and Millero, [11]).

The major contributors for water level decline are diversion of its main tributary “Jordan River” and chemical industry activities at the eastern and the western shores of the Dead Sea. The water level decline reported at an annual rate of more than one meter, which caused a shrunk in the total area by more than 35% in the last 30 years (Khlaifat et. al., [2, 6]; Frumkin and Elitzer, [12]; Khlaifat, [1]; Asmar and Ergenzinger, [13, 14]; and Gertman and Hecht, [15]). As consequences of the Dead Sea level decline two hazardous phenomena are resulting. The first phenomenon is the loss of groundwater due to the changes in the groundwater gradient and intrusion of freshwater that leads to dissolution of the salt brine that creates cavities (Salameh and El-Naser, [16, 17, 18]), while the second one is the sinkholes formation that causes damages for the agricultural land and development of the area on the shores of the Dead Sea (Salvati and Sasowsky, [19]; Abelson et al. [20]). It was found that the groundwater discharge rate to the Dead Sea increases with the receding base level, which overall results in an increase in the hydraulic gradient and seaward migration of brine/freshwater interface (Kiro et al. [21]; Salameh and El-Naser, [16, 17, 18]). Historically, the water flow in Jordan River has been decreased from 1.5 billion cubic meters annually in 1960s to less than 100 million cubic meters recently. The Jordan River catchment area is shared among Jordan, Syria, Lebanon, Israel, and Palestine. Dams, canals, and pumping stations were constructed in the catchment area countries to divert the water for irrigation and drinking purposes. Even the water quality in the Jordan River has been deteriorated due to the remaining brackish water flow and discharging of sewage.

The proposed Red Sea–Dead Sea Conduit would provide soundly environmental solution to save the Dead Sea and its ecosystem through eliminating the impact of the level decline and providing a continuous flow of Red Sea water. This project will provide drinking water and electricity to Jordan and the riparian countries of the Dead Sea, and will, sustain and raise the Dead Sea water level. During the filling period of the project, to raise the Dead Sea level, the salinity of the upper water layer will decrease. Also, the composition of this layer is expected to change. When filling process is over (steady state regime) evaporation will be compensated by Red Sea water discharge and the upper water layer salinity will start to increase.

The length of the proposed conduit is about 180 km and runs as a Canal from Red Sea to Dead Sea inside Jordanian territories only (Sarah and Fine [22]). The inflow of Red Sea water or concentrated desalination rejects (after water desalination) into the Dead Sea will have major impacts on its dynamics, physical, chemical, and the whole biological ecosystem. Water body stratification would be resulted due to the large surface flow (Beyth et al. [23]).

## 2 Dead Sea – Red Sea water mixing, experimental site and sampling

Mixing of Dead Sea water which is high in calcium (Ca ≥18 g/L) with Red Sea water that has high concentrations of sulfate (SO_4_^−2^ ≥ 3 g/L) would promote the natural formation of gypsum (CaSO_4_.2H_2_O) precipitate (Katz et al. [24], Reznik et al. [25, 26, 27]). Moreover, it was found that Halite (NaCl) began precipitating in 1983 as a consequence of increase in the Dead Sea water salinity and sustained constant precipitation rate since then (Steinhorn, [28]), (Gavrieli et al. [29]; Gavrieli, [9]).

The Dead Sea water salinity is a function of evaporation and the atmospheric relative humidity. It was found that Dead Sea surface water salinity increased from 225 g/kg to 279 g/kg during the period from late 1950s–1980s (Levy [30]). The reported Dead Sea salinity in the current study reached little above 325 g/kg. Recent studies showed that Dead Sea evaporation rate varied from 1.05 to 2.0 m/year for the current salinity (Stanhill [31]; Alpert et al [32]; Salameh and El-Naser [16] and Lensky et al. [33]). These values which based on water and heat balance calculations were in contrary with other studies that estimated the evaporation rate at the Dead Sea from 1.30 to 1.54 m (Al-Weshah, [34]).

Dead Sea hydrography was distinguished by two periods meromictic and holomictic in a long term monitoring study extended for 9 years from 1992 to 2000 (Gertman and Hecht, [15]). The meromictic period prolonged from 1992 to 1995, while the holomictic period from 1996 to 2000. The study described throughout the nine years the changes in both temperature and salinity in Dead Sea water body. Other investigations have described specific characteristic of the Dead Sea for shorter periods (Frumkin and Elitzur, [12]; Oren et al., [4]; Sinder et al., [35]). Moreover, as the Dead Sea brine is the raw material for the existing chemical industries, it was assessed for its physical and chemical properties for a period extended for 22 years from 1987 to 2008 (Khlaifat et. al., [36]). The proposed water conduit project is expected to have a direct impact on the Dead Sea water salinity and other water properties. The Red Sea water and/or desalination plant reject discharge point location that flow into Dead Sea would have a vital effect on the surface water salinity (Abu Qdais, [37]). Consequently, chemical industries located at both sides of Dead Sea will be adversely impacted. After this intensive literature review of the available data on properties of Dead Sea brine, the researchers concluded that there is still need for more basic data on a longer study period. Therefore, in the present work the physical properties of the mixing water scenarios were carried out for one year.

### 2.1 Experimental site

**Figure 1:**
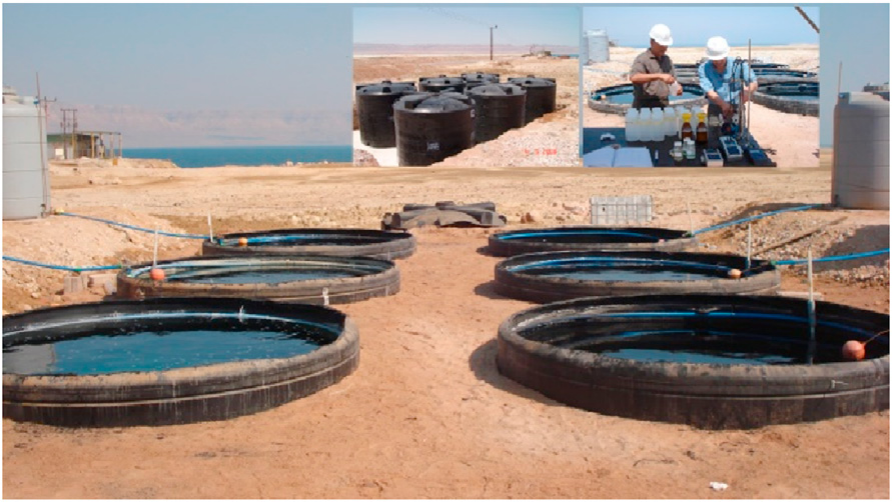
Experimental site.

This pilot project study used well-controlled mixed water ponds to investigate the interaction between Red Sea and Dead Sea waters. Experiments were conducted on the Dead Sea ground level. The experimental site was located next to Arab Potash Company point of intake about 100 m south of the Dead Sea shores. The site consists of six cylindrical tanks made from high density polyethylene (HDPE) material (diameter = 3 m and height = 3.5 m) buried for more than 3 m in the ground and open from the top as shown in Figure 1.

Four tanks (1–4) were filled with an approximately 25 m^3^ of a mixture of Dead Sea and Red Sea waters. The source of the Dead Sea water was from the pipeline that feeds the evaporation ponds of the Arab Potash Company. On the other hand the Red Sea water was transported via water tank trucks from the Gulf of Aqaba.

Before mixing the two water bodies, large particles in the Red Sea water were removed by filtration through a filter. Part of the filtered Red Sea water was concentrated by evaporation, in a water pool, to about twice its original salinity and mixed with the Dead Sea water in tank number 5 with the volume content shown in Table 1. Tank number 6 was filled only with Dead Sea water and used for benchmarking (Khlaifat et. al. [2, 5, 6]).

**Table 1:** Dead Sea (DS) and Red Sea (RS) water content (vol. %) in different tanks.

Tank Number	RS Water	DS Water	Concentrated Red Sea water
1	10	90	
2	20	80	
3	30	70	
4	40	60	
5	-	85	15
6	-	100	

During the monitoring period water level controller is used to maintain the mixed water level in each tank at a fixed height. Red Sea water was added to the top of tanks 1–4, concentrated Red-Sea water was added to the top of tank 5, and tank 6 surface level was kept constact by adding Dead Sea water. This water addition was done to compensate for water losses by evaporation. It was controlled by level control system, which consists of a float located at the mixed water-air interface, thus allowing more or less water to flow into each of the experimental tanks from the feed tanks. Each large tank is connected with a two cubic meter feed tank.

No external force is applied to mix the waters in the tanks. The two waters are mixed by natural mass transfer between lighter and denser sea waters in a system of fixed volume that is affected by seasonal variations. Diffusion, natural convective mass transport, and the motion of the interface (between each tank water surface level and atmosphere) upon evaporation and dissolution were the main modes of mixing.

### 2.2 Sampling

Water samples were collected from different water tanks (5 tanks of mixed waters and one tank of Dead Sea water only). During the winter, rain samples were collected as well. A collection time interval was adequate to monitor a variety of changes in salt concentration, temperature and rainfall. As long as the experimental work lasted for one year, having a two weeks' time interval for sampling was enough to capture different occurring phenomena caused by mixing. Samples for analysis were collected, from each tank, at different depths from the surface (surface level or top (up to 0.5m), middle (1.5 m), and bottom (2.5 m)) respectively using a close-bottle sampler.

Collected samples were kept away from light, even during transportation to the laboratory. Samples were stored in dark conditions at 4 °C. Water samples collected in clean polyethylene bottles (1.725 L) were divided into eight equal portions of 200 ml that were used for the analysis of anions, cations, physical parameters, chemical properties, and heavy metals.

### 2.3 Measurements and analyses

All analyses were conducted, right after the samples being collected, in the laboratories of Prince Faisal Center for Dead Sea, Environmental and Energy Research (at Mutah University) and Arab Potash Company laboratories. The collected samples were analyzed and investigated for different physico-chemical parameters, salt types and microbial effects. This paper discusses the results of physical properties measurement and analysis only, namely: temperature, pH, dissolved oxygen (DO), density, salinity, and viscosity. Interrelationships between these properties were investigates as well.

As long as Arab Potash Company's point of intake pumps is located at about 20m below Dead Sea level, then it is important to know the changes in the mixture physical properties at the bottom of each of the six tanks throughout the monitoring year.

Mixed water temperature is a key measurement obtained, in situ, by sensors located at different depths and verified using a conventional thermometer measurement.

The acidity, pH, of the mixed water was measured using a pH meter in situ right after collecting the water samples. The density of the collected samples was measured in situ using a hydrometer.

The salinity was measured in situ and in the lab too. Hydrometer was used to measure the specific gravity of the collected mixed water samples and then it was converted to salinity. Lab measurement of salinity followed the standard method for examination of water and wastewater for chloride amount in the mixed water samples (APHA, [38]). Salinity value was double checked for few samples using gravimetry by taking the weight of the total dissolved solids per a given volume of mixed water.

Dissolved oxygen was measured in situ using DO meter. The viscosity of the collected mixed water samples had been measured in the lab by master viscometer.

## 3 Results and discussions

### 3.1 Temperature

**Figure 2:**
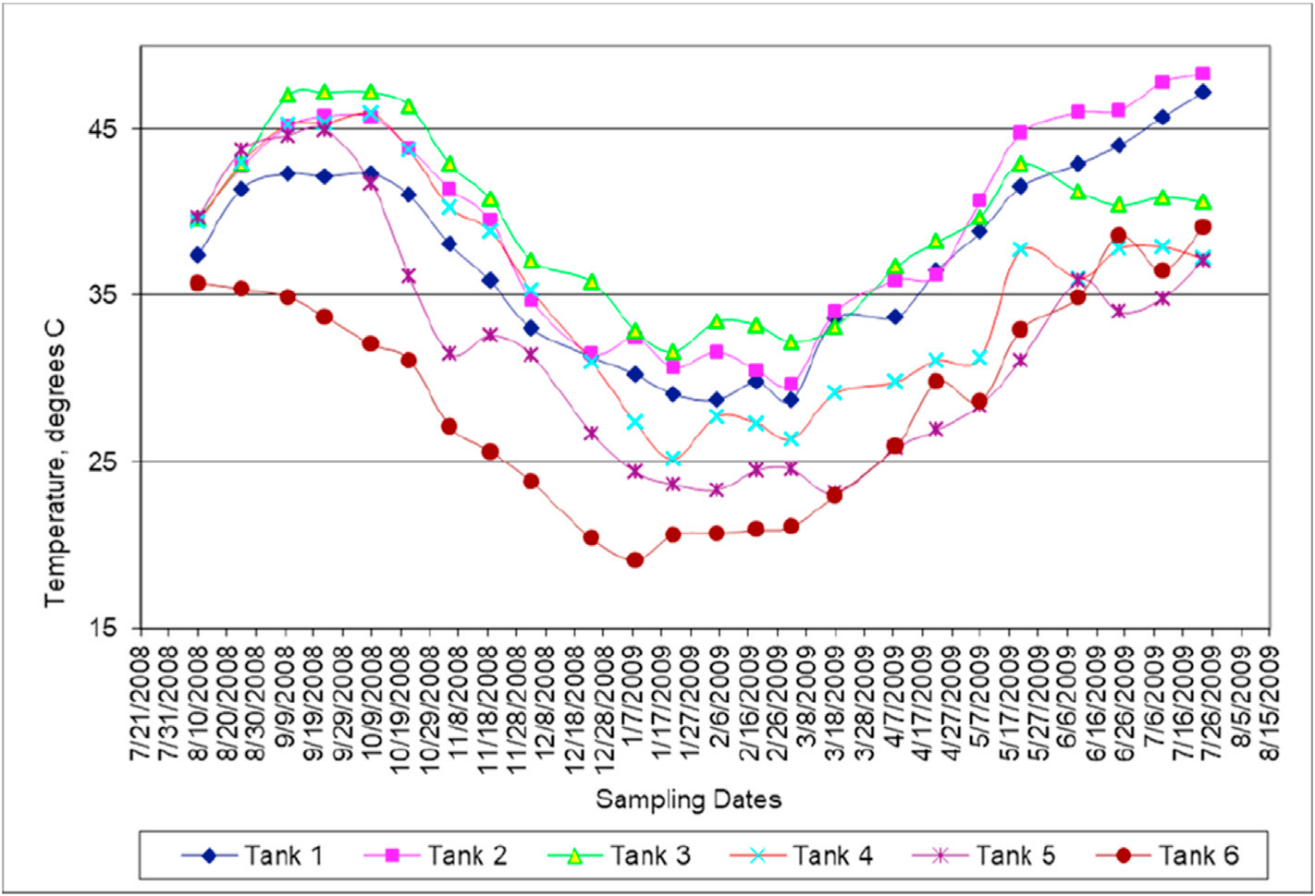
Temperature variation with time.

Figure 2 shows temperature variation at the bottom of each of the six tanks throughout the year.

It is well known that temperature contributes to all, mixed water, physical and chemical properties differences. The temperature profiles show variations in all tanks, Figure 2. The temperature changes depend on the climate conditions, highest temperatures were above 46 °C during the summer season, while during the winter season the temperature touched 17 °C. Likewise, after six month from monitoring the temperature profile, the rejected brine tank-5 had a similar temperature behavior like the DS water tank-6 temperature variation. While, all other tanks showed temperatures higher than either the DS water tank (tank-6) or the rejected brine tank (tank-5). The results of temperature profile explain the proportionality relationship between the water temperature and density. Lower temperatures in tanks 5 and 6 could be attributed to lower stratification level compared to other tanks (1–4). It will be shown later that continuous rise in temperature results in increasing both density and salinity.

### 3.2 Acidity (pH)

The water acidity which can be expressed by the pH value plays a vital role for the aquatic life. Most of marine organisms live in a pH range of 6.5–9.0, though some of them can live in ocean water with pH levels outside of this range. Therefore, pH value was monitored during the study period in the mixing water tanks. Additionally, the water acidity controls the trace metals solubility and mixed water toxicity. Also, the rate and products of chemical reactions occurring in some of the tanks depend on the acidity of the mixed water.

**Figure 3:**
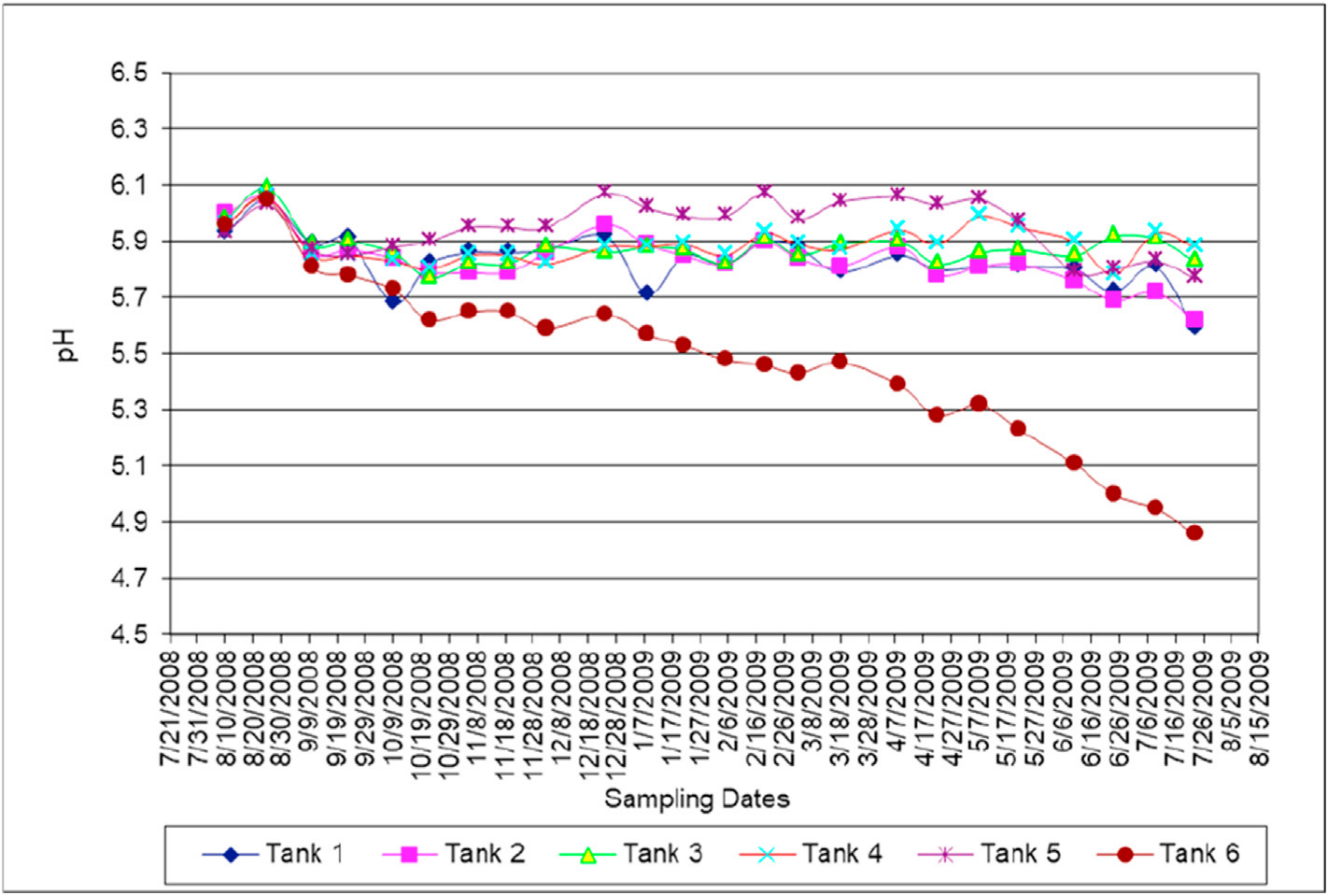
Acidity (pH) variation with time.

Figure 3 shows that the acidity of mixed water in all tanks is less than 6.1, which means that the environment is not perfect for aquatic creatures but might be good for some kind of bacteria. Mixed water acidity values range from 5.6 and 6.1 in all tanks excluding tank number 6 that contains Dead Sea water only. In tank 6 the pH reached a value of 4.8 after one year which restricts biological availability in this tank. The pH value was reported 5.9 for Dead Sea in 1977 (Ben-Yaakov and Sass, [39]). The decline in pH value in tank number 6 might be attributed due to the precipitation processes and formation of halite.

It was observed that the water acidity decreases with the increase in water temperature during the last 6 months of the experiments (Figure 2). The acidity variation does not mean certainly that water becomes more acidic at higher temperatures. But, the mixed waters solution became more acidic due to an excess of hydrogen ions over hydroxide ions.

### 3.3 Dissolved oxygen (DO)

Oxygen can enter the tanks via two different sources: 1) the main mechanism is atmospheric diffusion where oxygen in the air is absorbed by surface water due to a difference in oxygen concentrations; 2) continuous input of Red Sea water (tanks 1 to 4) and rejected brine (tank 5).

Both dissolved oxygen and temperature of the mixed water bodies are affected by seasonal weather variations and the physical properties of mixed water. DO level is directly related to how much aquatic life mixed water tanks can support.

**Figure 4:**
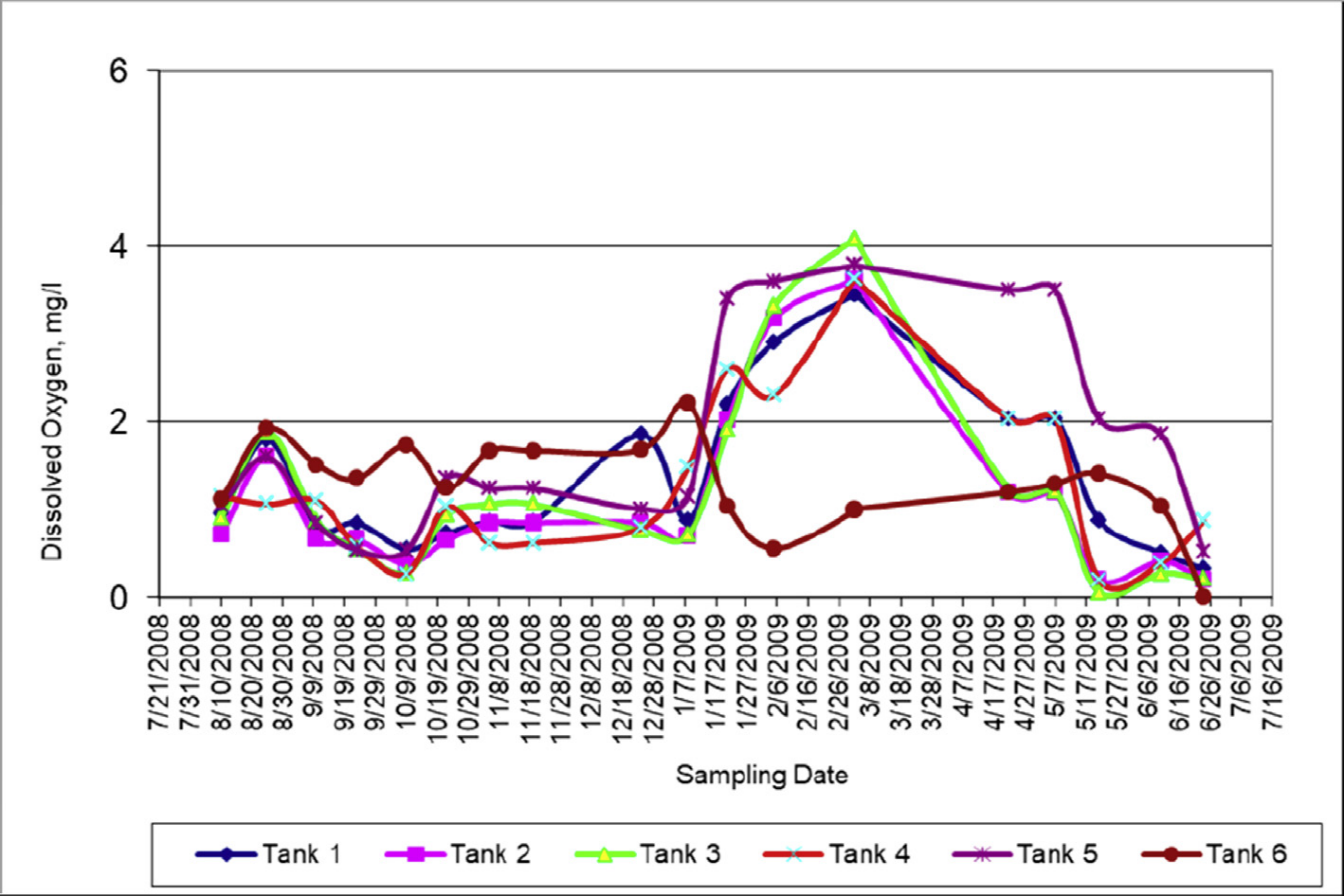
Dissolved oxygen variation with time.

It is clear from Figure 4 that the concentration of dissolved oxygen throughout the year is too low (less than 0.004 g/l), that makes it impossible to sustain aquatic life at the bottoms of all tanks. Some reasons for the low DO level could be chemical and biological oxygen demands. It was found that mixed water in all tanks except tank number 6 was stratified, which means that the hypolimnion receives little oxygen from atmospheric diffusion. Moreover, continuous feed streams come from feeding tanks and have only minimal impacts on the oxygen content of larger mixing tanks. Thus, the mixed water at the bottom of the tanks receives very little dissolved oxygen during summer thermal stratification but a little more during the winter time (see Figure 4). Moreover, DO levels for Dead Sea water column was reported at 0.0014 g/L during the period 1987–1989 (Shatkay, 1991 [40]; Shatkay et al., 1993 [41]). These values are comparable to this project/study findings.

### 3.4 Mixture density

**Figure 5:**
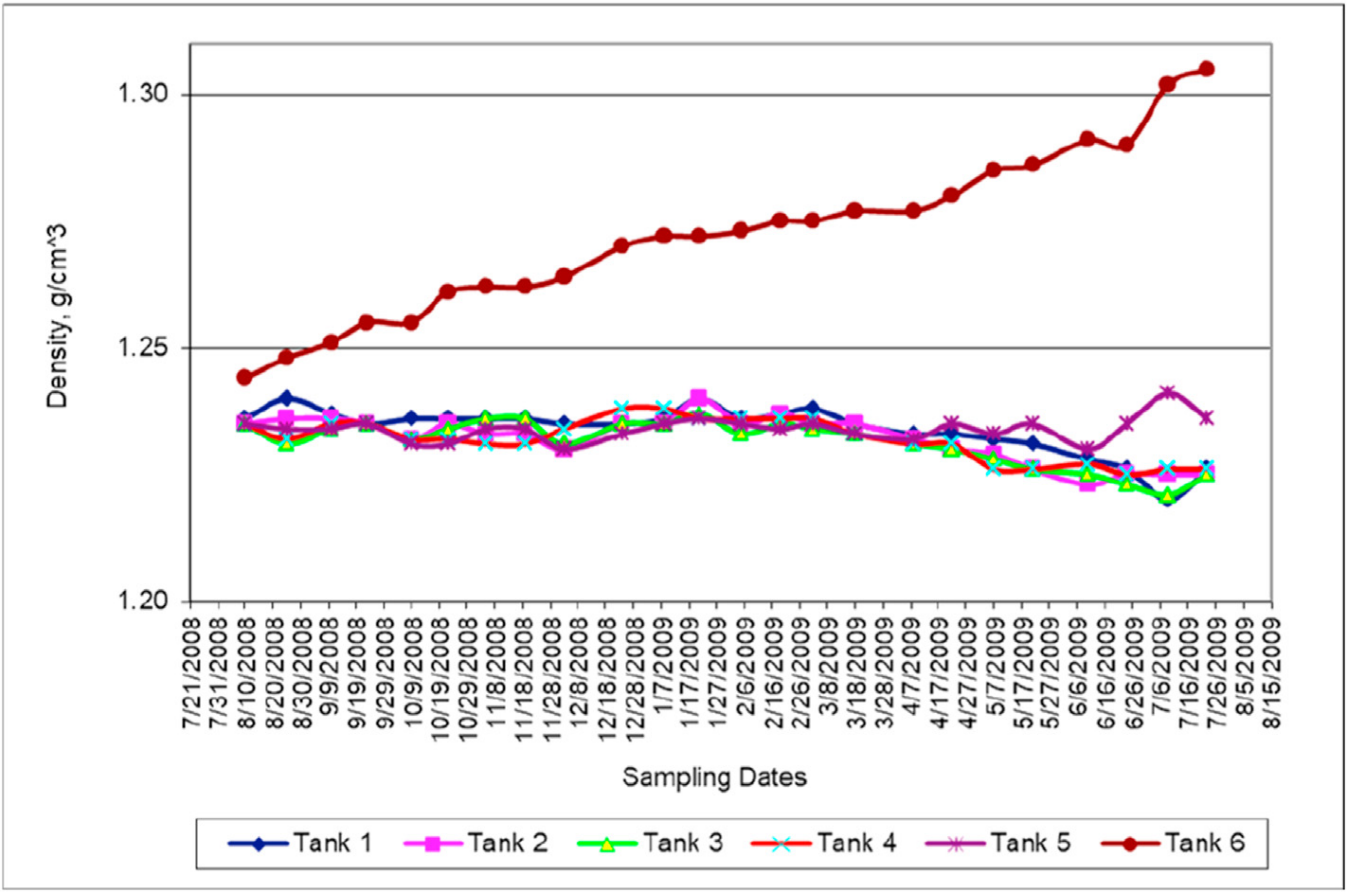
Density variation with time.

**Figure 6:**
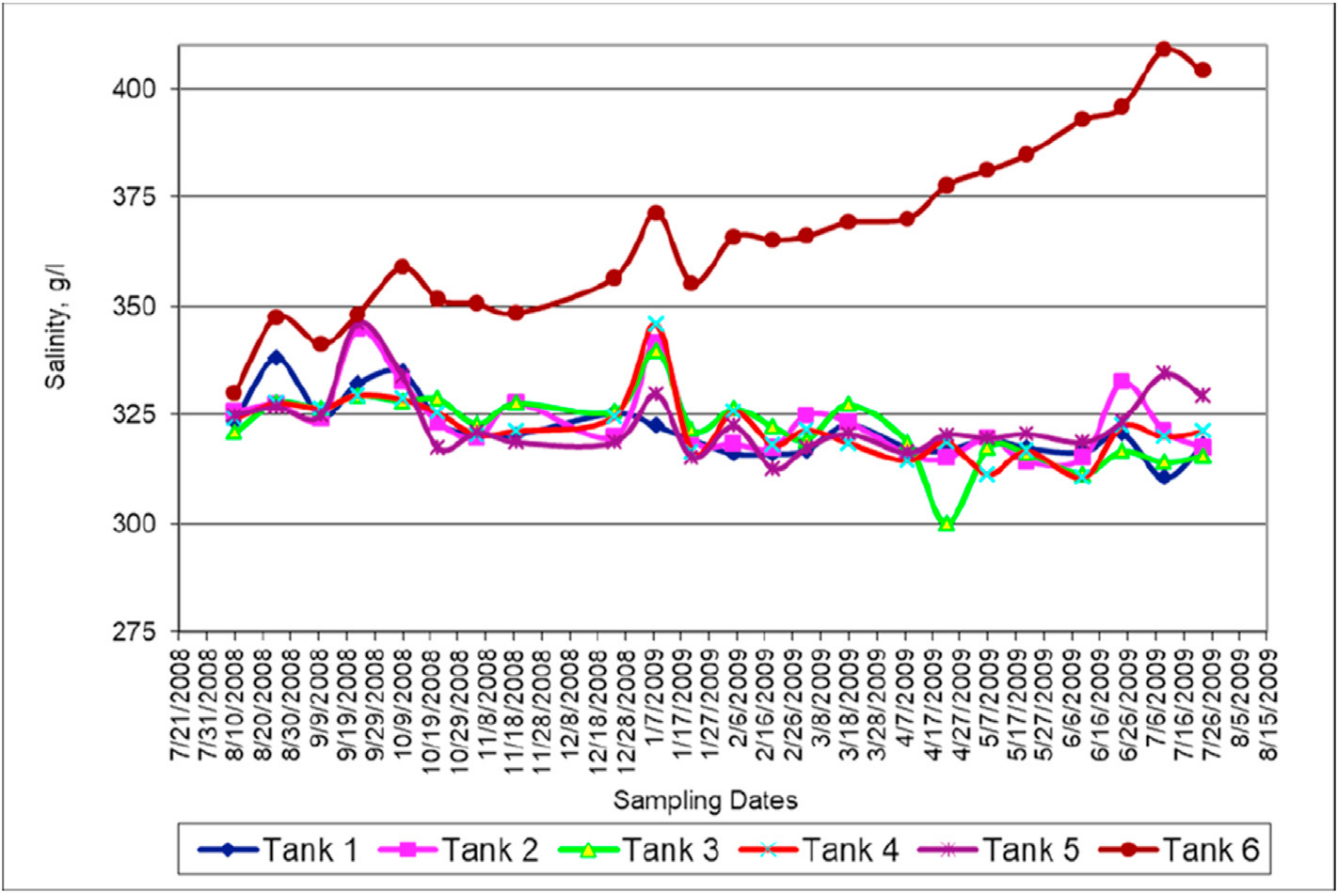
Salinity variation with time.

Temperature variations and dissolved substances contribute to minor density differences in different tanks. The density of Dead Sea water in tank 6 was increasing in a similar salinity trend increase. This indicates that the most important changes of mixed water physical properties in all tanks are the changes in the relationship among water density (Figure 5), salinity (Figure 6) and temperature (Figure 2). Laboratory tests and analyses performed included: temperature, density and salinity (APHA test procedure [38]).

### 3.5 Salinity (TDS)

Salinity is a measure of the amount of salts in the mixed water tanks. Red Sea water was considered as a freshwater where the term “total dissolved solids” (TDS) was used instead of “salinity”.

The Dead Sea water owes its high salinity due to a combination of dissolved ions of different salts such sodium chloride (NaCl), potassium chloride (KCl), magnesium chloride (MgCl_2_) and calcium chloride (CaCl_2_). The high concentration of dissolved ions results in increasing the salinity as well as conductivity of the Dead Sea water (tank 6). Salinity variation with time (Figure 6) has similar trends in all tanks to the trends observed for density variation and with time (Figure 5) this is due to the fact that these two properties strongly depend on salt contents. Salinity, likewise density, rise is driven by increased rate of evaporation and precipitation of sodium chloride (salt) from the saturated brine [9].

### 3.6 Viscosity

**Figure 7:**
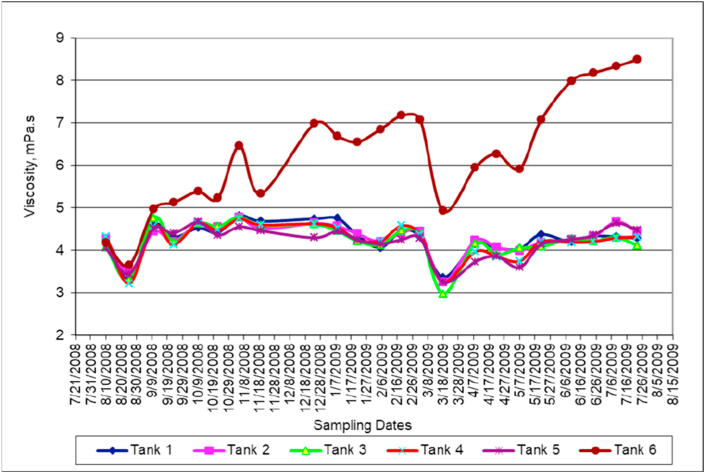
Viscosity variation with time.

It is clear from Figure 7 that the viscosity in all tanks but tank number 6 is decreasing. The viscosity of the mixed water in tank 6 was increased significantly. High viscosity values result in lowering diffusion rate.

It is well known fact that both viscosity and surface tension of viscous fluid increase as the temperature decrease. This fact is violated here with respect to the mixed water in tank 6, this is attributed to an increase in both salanity and density.

Both the viscosity (Figure 7) and density (Figure 5) of the mixed water samples collected from different tanks are functions of salinity (Figure 6), temperature (Figure 2) and pressure. However, for the depth range of our experiments, the dependency of pressure is negligible. Comparison between salinity (Figure 6) and viscosity (Figure 7) shows a direct proportionality.

## 4 Conclusion

This study presents the first experimental efforts in establishing a database for the physical properties of mixed Dead Sea and Red Sea waters that were conducted on the southern shore of the Dead Sea throughout 12 months to monitor all changes induced by seasonal variations. All the monitored physical properties, temperature, acidity (pH), dissolved oxygen, density, salinity and viscosity, had different degrees of changeability on timescales of months as shown in the results and discussions part of the paper.

None of the measured properties of mixed waters in tanks 1 to 5 tends to behave exactly like Dead Sea water (tank 6). The results showed that diluting the Dead Sea water, by Red Sea water and rejected brine, affects all its physical properties significantly. Physical properties measured when the rejected brine was added to the Dead Sea brine (tank 5) where the closest to Dead Sea brine properties (tank 6). Based on the industrial needs for the Dead Sea brine with its current physical properties, it is not recommended to add Red Sea water directly to the Dead Sea. The effect of adding rejected brine to the Dead Sea is minimal.

The obtained experimental results showed that natural convection influenced the mixing effect drastically. The observed changes in the studied physical properties confirm that convective flux is much higher than diffusive flux in the top layers (Red Sea water) of the tanks and the opposite is true for the lower layers (Dead Sea brine). It is not clear which flux (convective or diffusive) is dominating across the interface, this can be investigated further by chemical properties study and by numerical modeling and simulation which are beyond the scope of this paper.

## Declarations

### Author contribution statement

A. Khlaifat, M. Batarseh: Conceived and designed the experiments; Performed the experiments; Analyzed and interpreted the data; Contributed reagents, materials, analysis tools or data; Wrote the paper.

K. Nawayseh: Conceived and designed the experiments; Performed the experiments; Analyzed and interpreted the data; Contributed reagents, materials, analysis tools or data.

J. Amira, E. Talafeh: Conceived and designed the experiments; Performed the experiments; Contributed reagents, materials, analysis tools or data.

### Funding statement

This work was supported by the Arab Potash Company and Mutah University.

### Competing interest statement

The authors declare no conflict of interest.

### Additional information

No additional information is available for this paper.
